# Cardiorespiratory Responses to Exercise in Hypobaric versus Normobaric Hypoxia: A Randomized, Single-Blind, Crossover Study

**DOI:** 10.1249/MSS.0000000000003578

**Published:** 2024-10-04

**Authors:** GIOVANNI VINETTI, RACHEL TURNER, ANNA TABONI, SIMON RAUCH, PAOLO MARIO ENRICO SERAGLIO, NIKOLAUS NETZER, GIACOMO STRAPAZZON, HANNES GATTERER

**Affiliations:** 1 Institute of Mountain Emergency Medicine, Eurac Research, Bolzano, ITALY; 2 Department of Molecular and Translational Medicine, University of Brescia, ITALY; 3 Department of Anaesthesia and Intensive Care Medicine, Hospital of Merano (SABES-ASDAA), Merano (BZ), ITALY; 4 Department of Medicine, University of Padova, ITALY; 5 SIMeM Italian Society of Mountain Medicine, Padova, ITALY

**Keywords:** EXERCISE PERFORMANCE, F_I_O_2_, HEART RATE VARIABILITY, HIGH ALTITUDE, HYPOBARIC CHAMBER, TERRAXCUBE

## Abstract

**Purpose:**

There is controversy whether there are meaningful physiological differences between hypobaric (HH) and normobaric hypoxia (NH). This study aimed to compare the cardiorespiratory responses to acute HH and NH under strictly controlled conditions. We hypothesized no differences at rest and during submaximal exercise, whereas during maximal exercise, a higher maximal ventilation (V̇_Emax_), peripheral oxygen saturation (SpO_2_), and maximal oxygen consumption (V̇O_2max_) in HH than in NH.

**Methods:**

In a randomized, single-blind, crossover design, eight young healthy subjects (three females) were studied in an environmental chamber in which either the barometric pressure (HH) or the inspired oxygen fraction (NH) was reduced to the equivalent of ~4000 m altitude. Measurements were taken at rest, and during submaximal (moderate and high intensity) and maximal cycling exercise.

**Results:**

All resting parameters were similar between HH and NH, except for a lower root mean square of the successive R-R interval differences in HH (*P* < 0.05). SpO_2_ was 2% higher in HH at all exercise intensities (*P* < 0.05). During submaximal exercise, minute ventilation was similar between HH and NH. However, HH yielded a 7% lower tidal volume during moderate-intensity exercise (*P* < 0.05) and a lower respiratory exchange ratio during high-intensity exercise (*P* < 0.01). V̇_Emax_ and V̇O_2max_ were 11% and 6% higher in HH, respectively (*P* < 0.01 for both). SpO_2_ at maximal exercise was positively correlated with V̇_Emax_, V̇_Emax_/V̇O_2max_, and V̇O_2max_.

**Conclusions:**

The higher V̇O_2max_ found in HH than in NH can be attributed to the higher V̇_Emax_ counteracting desaturation at maximal exercise. Conversely, submaximal SpO_2_ improved in HH through mechanisms other than increased ventilation. These findings are likely due to respiratory muscle unloading in HH, which operated through different mechanisms depending on exercise intensity.

Ambient hypoxia is characterized by a reduced partial pressure of O_2_ (P_I_O_2_) and can result from a reduction in either barometric pressure (*P*_B_), termed hypobaric hypoxia (HH), or the fraction of oxygen in the inspired gas mixture (F_I_O_2_), termed normobaric hypoxia (NH) (1). Whether HH and NH elicit the same physiological responses is controversial (2–7). At rest, lower peripheral oxygen saturation (SpO_2_) in HH compared with NH was reported (8–11), along with a lower (8,12) or similar (10) minute ventilation ( ${\dot{V}}_{E}$). Heart rate (HR) was reportedly higher in HH (8,9) or similar (10), whereas HR variability (HRV) was either lower in HH (9) or again similar (11). Adding to the controversy, others found no physiological differences at all between the two conditions at rest (1). During submaximal exercise, some studies reported lower SpO_2_ and higher HR in HH compared with NH (13,14) or lower ${\dot{V}}_{E}$ (15), results that remain either unconfirmed or only partially confirmed by others (15–18). At maximal exercise, theoretical models (19) and experimental evidence (20) suggest a higher maximal oxygen consumption ( $\dot{V}O_{2\max}$) in HH compared with NH, due to improved maximal ventilation ( ${\dot{V}}_{\text{Emax}}$) because of the lower air density. However, meta-analytic findings did not detect a statistically significant difference in $\dot{V}O_{2\max}$ between acute HH and NH (21). These inconsistent outcomes have been attributed to differences in the magnitude and/or duration of hypoxia (6,22), inspired carbon dioxide partial pressure, humidity and temperature (6,23), and spontaneous daily *P*_B_ fluctuations at high altitude (5).

Less discussed confounding factors are related to denitrogenation kinetics, the “alveolar gas equation effect” and the use of respiratory valves and tubing. Contrary to NH, in HH the reduced inspired N_2_ partial pressure promotes tissue denitrogenation, with excess tissue N_2_ being washed out through the lungs (24). This transient excess N_2_ exhalation and dilutes the other alveolar gases, so that alveolar O_2_ and CO_2_ partial pressures (P_A_O_2_ and P_A_CO_2_) are transiently reduced relative to NH (25), a finding that has been consistently observed during and immediately after decompression (8,10). After N_2_ equilibration, the alveolar gas equation applies,^1^ where the more the respiratory exchange ratio (RER) deviates from 1.0, the more the alveolar O_2_ mass balance depends on F_I_O_2_ (26), resulting, *ceteris paribus*, in higher P_A_O_2_ in HH than in NH the more the RER is <1.0, and vice versa (27). Finally, breathing through a three-way valve and the associated tubing has often been performed to administer the normobaric hypoxic mixture and/or to prevent CO_2_ build-up in the normobaric/hypobaric chamber (8,10,14) or to connect the subjects to a mixing chamber respirometry system (16). However, this setup increases breathing resistance and dead space (28) and may represent a confounding factor on ventilatory mechanics of a greater magnitude than the actual difference between HH and NH itself.

The primary aim of this study was to confirm or challenge current notions on the differences in cardiorespiratory responses between acute HH and NH at rest and during exercise by using a high-quality design. We controlled for the aforementioned confounders by allowing free breathing of P_I_O_2_-matched normobaric and hypobaric ambient air equivalent to ~4000 m altitude in a randomized, single-blind, crossover fashion. We hypothesized maximal exercise SpO_2_, ${\dot{V}}_{\text{Emax}},\text{and}\;\dot{V}O_{2\max}$ to be higher in HH than in NH, whereas we did not expect any difference during rest and submaximal exercise.

## METHODS

### Participants

Eight healthy individuals (three females) aged 31 ± 5 yr (range, 23–39) who were physically fit (176 ± 6 cm, 69 ± 9 kg, body mass index 22 ± 3 kg m^−2^ (range, 18–27), $\dot{V}O_{2\max}$ 55.0 ± 6.6 mL·kg^−1^·min^−1^ (range, 44.7–66.4) gave written informed consent to participate and completed the study. They were all nonsmokers, had no history of systemic disease, and resided at altitudes <1300 m. They were instructed to abstain from heavy exercise and to match nutrition and fluid intake during the 48 h (including a light meal 2 h before testing) and to avoid high-altitude (>2500 m) exposure during the 4 wk preceding testing sessions. The study was conducted in accordance with the Declaration of Helsinki and was approved by the Ethics Committee for Clinical Trials of the Autonomous Province of Bolzano (No. 92-2020).

### Interventions

Tests were performed in a large (12×6×5 m), well-ventilated (inspired CO_2_ fraction <0.1%) environmental chamber capable of controlling either *P*_B_ or F_I_O_2_ of the ambient air (terraXcube, Bolzano, Italy; 250 m above sea level). Three identical sessions were performed: the first in normoxia for familiarization, followed by a session in HH or NH after 3–10 d. After a 2-wk break, tests were performed in the remaining condition (NH or HH). The order was randomized, and the participants were blinded to the order. In the first hypoxic session, *P*_B_ or F_I_O_2_ was set to 462.3 mm Hg (HH) or 13.0% (NH), respectively, whereas in the subsequent session, F_I_O_2_ or *P*_B_ was individually set to match the P_I_O_2_ of the first session, accounting for daily variations in atmospheric pressure. Considering the saturated water vapor pressure at 37°C (47 mm Hg) (29), this resulted in a P_I_O_2_ of 89.5 ± 2.3 mm Hg in HH and 89.7 ± 2.0 mm Hg in NH (*P* = 0.579), corresponding to an equivalent altitude of 4006 ± 168 m with the model atmosphere equation (30). Temperature and humidity were kept constant at 21.0°C ± 0.1°C and 40% ± 3%, respectively.

After entering in the environmental chamber, the subject was placed supine on a bed, and during the next 20 min, the chamber either was progressively decompressed to the target *P*_B_ (HH) or was progressively flushed with N_2_ to reach the target F_I_O_2_ (NH). To ensure participant blinding, decompression and recompression phases were simulated at the beginning and the end of the NH session by means of low-amplitude (~40 mm Hg) decompression–recompression cycles. After reaching the target *P*_B_ or F_I_O_2_, subjects rested supine for an additional 25 min, with resting measurements recorded during the last 5 min. Then, after a 20-min break, they were seated on a cycle ergometer (E100; COSMED, Rome, Italy), saddle height was individually adjusted and recorded, and they were instructed to start pedaling at a self-selected cadence ≥80 min^−1^. Power output was initially maintained at 60 W for females and 80 W for males ( ${\dot{W}}_{60/80}$) for 10 min to ensure steady-state conditions, and then it was increased every minute by 20 W for males and 15 W for females until exhaustion (defined as inability to maintain pedaling cadence despite strong verbal encouragement). Maximal mechanical power output ( ${\dot{W}}_{\max}$) was calculated as the work rate of the last completed step plus the fractional duration of the last uncompleted step multiplied by the power increment (31). To verify the attainment of HR_max_ and $\dot{V}O_{2\max}$, after a 30-min recovery, a supramaximal verification bout at 105% of ${\dot{W}}_{\max}$ was carried out to exhaustion, preceded by a warm-up consisting of 4 min at 40% ${\dot{W}}_{\max}$ and 2 min at 70% ${\dot{W}}_{\max}$.

### Measurements

Throughout all sessions subjects wore an electrocardiograph, a fingertip SpO_2_ monitor on the nondominant hand (WristOx_2_ Model 3150 with 8000SM sensor; Nonin Medical, Plymouth, MN) and a face mask connected to a portable breath-by-breath gas exchange analyzer (METAMAX® 3B; CORTEX Biophysik, Leipzig, Germany) that was calibrated according to the manufacturer’s instructions. To ensure reliable SpO_2_ readings, the nondominant hand was kept warm with a loose-fitting surgical glove to avoid vasoconstriction, and participants were instructed to keep it still and to use only the contralateral hand to grip the ergometer’s handlebar. Oxygen uptake, carbon dioxide output ( $\dot{V}O_{2}$ and $\dot{V}{\mathrm{CO}}_{2}$, at standard temperature and pressure, dry air, stpd), respiratory exchange ratio ( $\dot{V}{\mathrm{CO}}_{2}/\dot{V}O_{2}$, RER), ${\dot{V}}_{E}$ (at body temperature and *P*_B_, saturated with water vapor, btps), tidal volume (V_T_), respiratory frequency (*f*_R_), and end-tidal gas partial pressures (end-tidal oxygen partial pressure (P_ET_O_2_) and end-tidal carbon dioxide partial pressure (P_ET_CO_2_) were recorded. Continuous arterial blood pressure was measured noninvasively by finger plethysmography (Finapres® NOVA; FMS, Amsterdam, the Netherlands) at rest only. Electrocardiogram and blood pressure waveforms were sampled at 1 kHz (PowerLab 16/35 and LabChart software; ADInstriments, Dunedin, New Zealand).

### Data treatment

Cardiorespiratory data were averaged over the last 5 min of the 25-min supine rest, the 10-min moderate-intensity exercise step ( ${\dot{W}}_{60/80}$), and the last 30-s of a fixed, high-intensity step of the incremental test, namely, 165 W for females and 200 W for males ( ${\dot{W}}_{165/200}$). Peak cardiorespiratory data were assessed as the highest 30-s average near the end of the incremental test and the supramaximal verification bout (indicated with the suffix “max” and “verif,” respectively). For comparison purposes, the predicted $\dot{V}O_{2\max}$ in hypoxia was calculated by the meta-analytic equation of Macinnis et al. (21), whose input variables are sea-level $\dot{V}O_{2\max}$ normalized per body mass ( $\dot{V}O_{2\max}/\mathrm{kg}$) and Model Atmosphere’s equivalent altitude (30). By means of the *CVRanalysis* 1.0 software (32), resting baroreflex sensitivity (BRS) was calculated with the sequence method (33) and resting HRV with time-domain and spectral analysis. Root mean square of the successive R-R interval differences (RMSSD), total power of the R-R spectrum (Ptot), spectral power of the very low-frequency (VLF; 0.04–0.15 Hz), low-frequency (LF; 0.15–0.40 Hz), high-frequency (HF, 0.15–0.40 Hz) band, and the LF/HF ratio were extracted. LF and HF were also expressed in normalized units (LFnu and HFnu), which represent their relative value in proportion to Ptot minus VLF.

### Statistics

Sample size was calculated to detect a 2% mean difference in SpO_2_, assuming a 2% SD of difference, a type I error of 0.05, and a statistical power of 0.8, resulting in *n* = 8 matched pairs. Normal distribution of the data was assessed by means of Shapiro–Wilk test and normal Q-Q plots and was rejected only for resting BRS, RMSSD, Ptot, and HF, which are presented as median (interquartile range), whereas the remaining resting data and all the exercise data are presented as mean ± SD. Resting data were compared between HH and NH by paired-sample two-tailed *t*-test if normally distributed or by Wilcoxon signed-rank test if not. Incremental exercise data were investigated with two-way ANOVA for repeated measures to assess the effect of condition (HH or NH) and exercise intensity ( ${\dot{W}}_{60/80}$, ${\dot{W}}_{165/200}$, and ${\dot{W}}_{\max}$) and their interaction. If Mauchly’s test rejected sphericity, the Greenhouse–Geisser correction was applied. In case of a significant effect of condition, or condition–intensity interaction, the Šidák multiple comparison test was performed between HH and NH of the same intensity, using a single pooled variance when sphericity was met, or computing individual variances for each comparison if sphericity was rejected. The effect size was determined by Cohen’s *d* (Wilcoxon’s *r* for nonnormally distributed data) and classified as follows: 0.2–0.4 small, 0.5–0.7 medium, and ≥0.8 large (34). The agreement between HR_max_ and HR_verif_ and $\dot{V}O_{2\max}$ and $\dot{V}O_{2\text{verif}}$ was assessed by Bland–Altman analysis. Linear regression analysis was applied to selected variables for which there could be a plausible physiological relationship. The level of significance was set at *P* < 0.05. The statistical package Prism 9 (GraphPad Software, La Jolla, CA) was used.

## RESULTS

**FIGURE 1:**
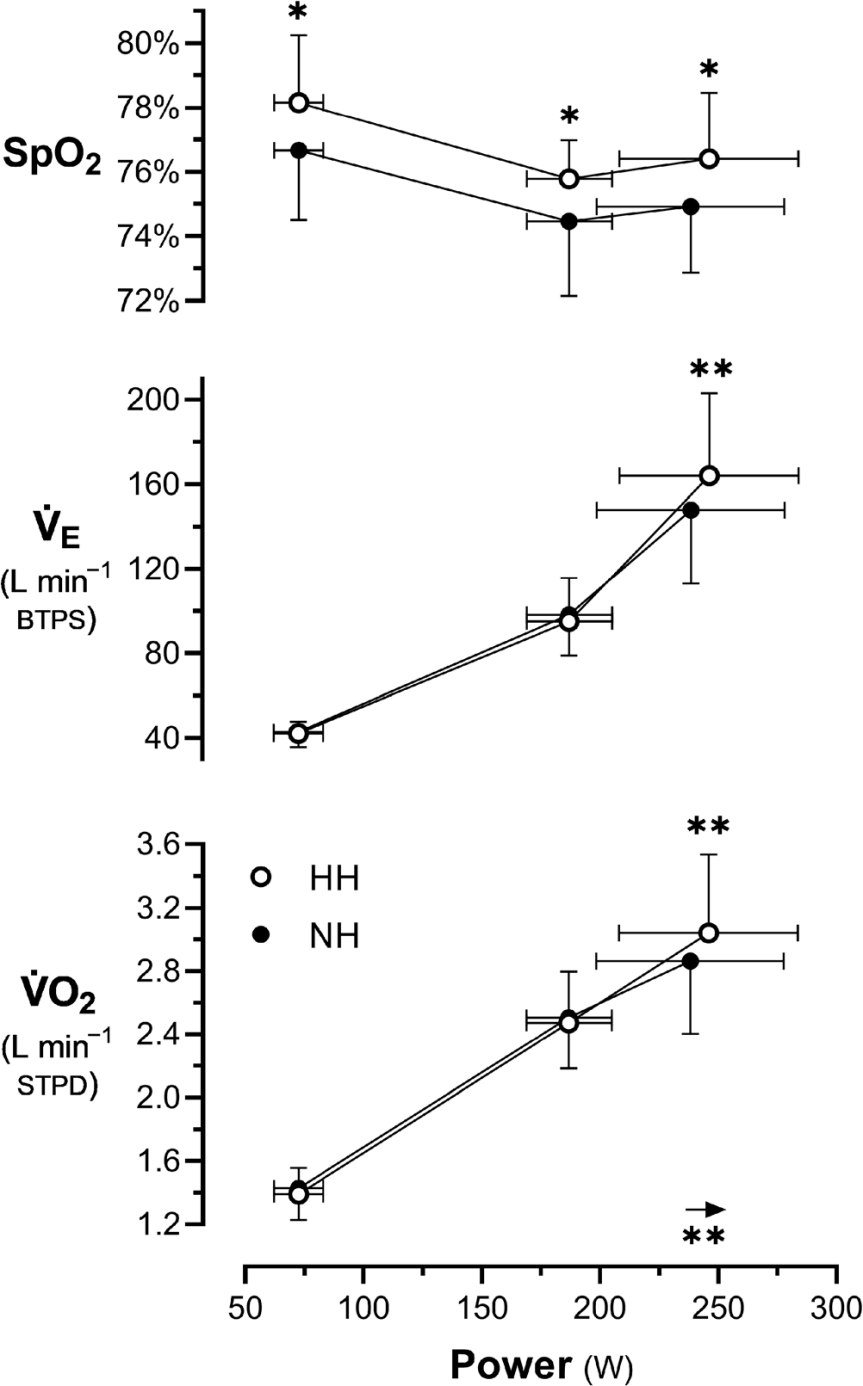
SpO_2_, ${\dot{V}}_{E}$, and $\dot{V}O_{2}$ as a function of absolute mechanical power output. Arrow indicates the increase in maximal power output in HH compared with NH. Open symbols, HH; closed symbols, NH. *P < 0.05, ** *P* < 0.01 versus NH.

**FIGURE 2:**
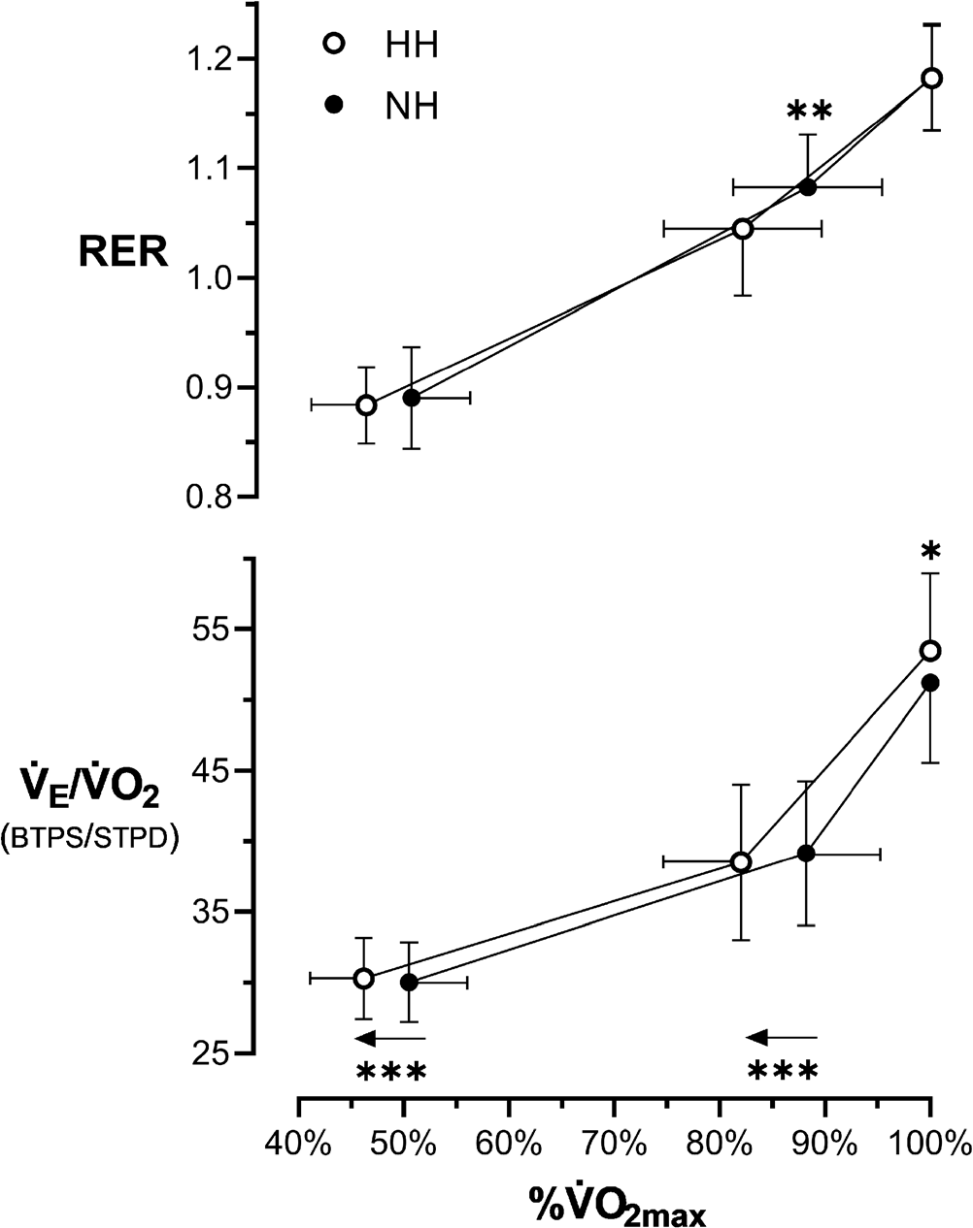
RER and the ventilatory equivalent for oxygen ( ${\dot{V}}_{E}/\dot{V}O_{2}$) as a function of percentage utilization of the condition-specific $\dot{V}O_{2\max}$. The arrows indicate the shift of the % $\dot{V}O_{2\max}$ in HH compared with NH. The RER points lie on the same relationship, while the ${\dot{V}}_{E}/\dot{V}O_{2}$ versus % $\dot{V}O_{2\max}$ curve is shifted leftward and upward in HH compared with NH. Open symbols, HH; closed symbols, NH. *P < 0.05, ** *P* < 0.01, and *** *P* < 0.001 versus NH.

**FIGURE 3:**
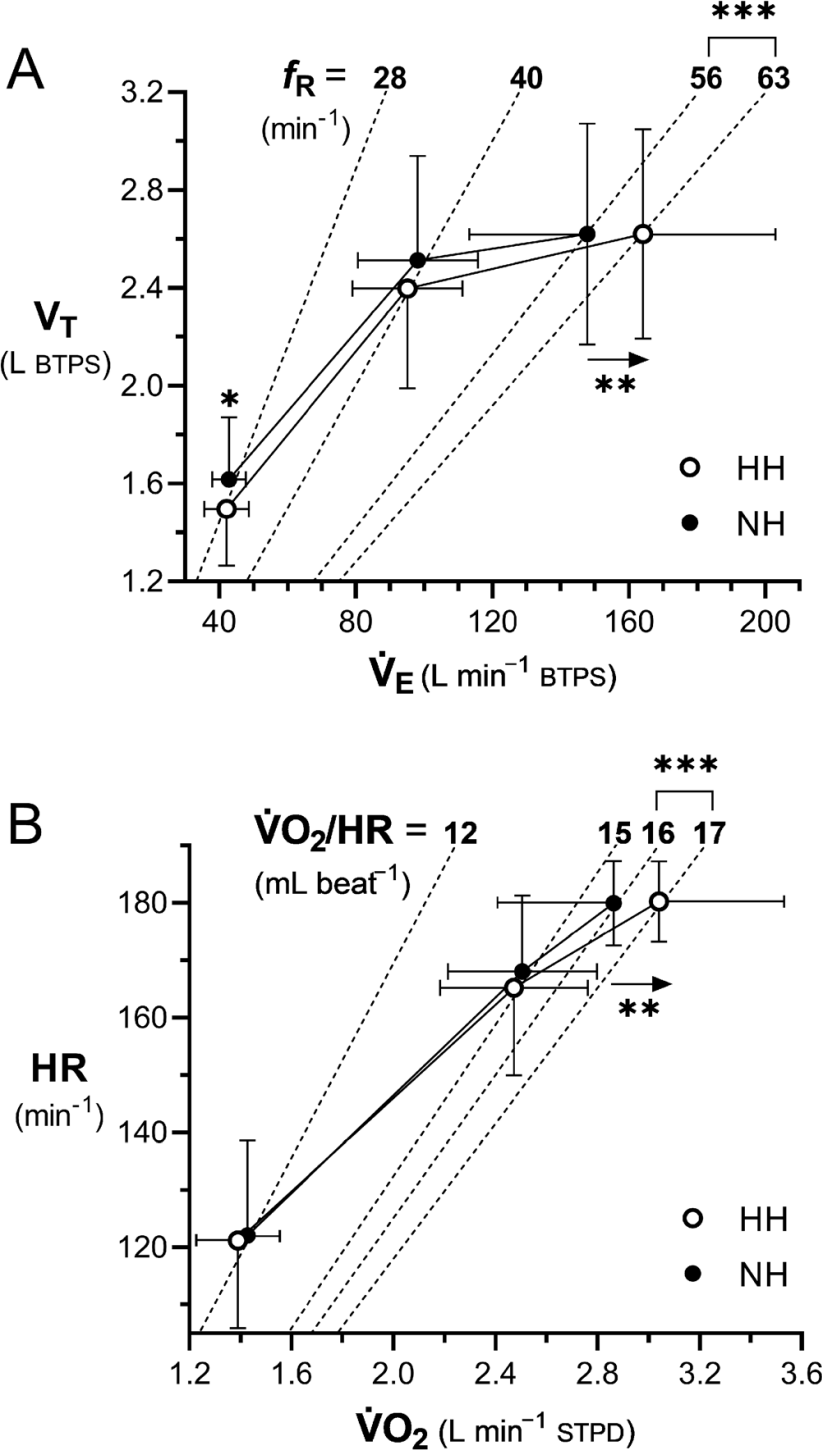
A, Relationship between V_T_ and ${\dot{V}}_{E}$. Dashed lines represent isopleths for the indicated respiratory frequency (*f*_R_), and arrows indicate the increase in ${\dot{V}}_{E}$ in HH compared with NH. B, Relationship between exercise HR and $\dot{V}O_{2}$. Dashed lines represent isopleths for the indicated $\dot{V}O_{2}/\mathrm{HR}$ ratio, and arrows indicate the increase in maximal $\dot{V}O_{2}$ in HH compared with NH. For all panels: open symbols, HH; closed symbols, NH. *P < 0.05, ** *P* < 0.01, and *** *P* < 0.001 versus NH.

No significant differences were found between HH and NH at rest except for RMSSD, which was higher in HH with a medium effect size (Table 1). During exercise, there was a significant effect of intensity on all investigated variables (*P* = 0.040 for SpO_2_, *P* < 0.0001 for all others). The effect of condition, condition–intensity interaction, and the pairwise comparisons between conditions at a given intensity are shown in Table 2. Although submaximal $\dot{V}O_{2}$ and ${\dot{V}}_{E}$ were similar between conditions, SpO_2_ was higher in HH at all intensities (~2% relative difference) and ${\dot{W}}_{\max}$, $\dot{V}O_{2\max},\text{and}\;{\dot{V}}_{\text{Emax}}$ were higher in HH (Fig. 1) (medium effect size for all). Additionally, V_T_ at ${\dot{W}}_{60/80}$ and RER at ${\dot{W}}_{165/200}$ were significantly lower in HH compared with NH (medium effect size for both). However, the relationship between RER and percent $\dot{V}O_{2\max}$ utilization was similar (Fig. 2). At maximal exercise, *f*_R_, ${\dot{V}}_{\text{Emax}}/\dot{V}O_{2\max}$, and P_ET_O_2_ were also significantly higher in HH compared with NH (medium effect size for all), P_ET_CO_2_ was lower (large effect size), whereas RER, V_T_, and HR_max_ were similar. As a consequence, the higher ${\dot{V}}_{\text{Emax}}$ in HH was obtained through a higher *f*_R_ (Fig. 3A), and the $\dot{V}O_{2\max}/{\mathrm{HR}}_{\max}$ ratio was higher in HH than in NH (small effect size) (Fig. 3B). The time to exhaustion of the supramaximal verification bout was 106 ± 16 s in HH and 112 ± 19 s in NH (*P* = 0.455). In both HH and NH, compared with the incremental test, the supramaximal verification bout did not show significantly different peak HR (178 ± 6 min^−1^ in HH, *P* = 0.130 vs incremental test, and 179 ± 7 min^−1^ in NH, *P* = 0.163 vs incremental test) and $\dot{V}O_{2}$ (3.019 ± 0.485 L·min^−1^ in HH, *P* = 0.458 vs incremental test, and 2.841 ± 0.481 L·min^−1^ in NH, *P* = 0.620 vs incremental test), with high agreement regardless of HH or NH (overall $\dot{V}O_{2\text{verif}}$ − $\dot{V}O_{2\max}$ bias −0.022 ± 0.099 L·min^−1^, 95% limits of agreement −0.216 to +0.172 L·min^−1^; overall HR_verif_ − HR_max_ bias −2 ± 3 min^−1^, 95% limits of agreement −7 to +4 min^−1^).

**TABLE 1:** Parameters at rest in NH and HH.

	NH	HH	*P*	Effect Size
SpO_2_ (%)	78.4 ± 2.6	79.2 ± 2.5	0.090	0.30
$\dot{V}O_{2}$ (L·min^−1^ stpd)	0.289 ± 0.044	0.291 ± 0.045	0.803	0.06
RER	0.81 ± 0.03	0.81 ± 0.02	0.241	0.28
${\dot{V}}_{E}$ (L·min^−1^ btps)	9.1 ± 1.7	8.8 ± 1.5	0.328	0.24
V_T_ (L btps)	0.60 ± 0.13	0.59 ± 0.11	0.612	0.12
*f*_R_ (min^−1^)	15.7 ± 4.1	15.4 ± 4.3	0.736	0.07
${\dot{V}}_{E}/\dot{V}O_{2}$ (btps/stpd)	31.5 ± 2.7	30.1 ± 2.6	0.142	0.56
${\dot{V}}_{E}/\dot{V}{\mathrm{CO}}_{2}$ (btps/stpd)	38.7 ± 3	37.2 ± 3.2	0.272	0.48
P_ET_O_2_ (mm Hg)	50.7 ± 2.1	50.7 ± 2.5	0.985	0.01
P_ET_CO_2_ (mm Hg)	32.7 ± 1.8	32.6 ± 1.9	0.898	0.03
HR (min^−1^)	59 ± 6	60 ± 5	0.283	0.23
SAP (mm Hg)	118 ± 6	116 ± 7	0.572	0.35
DAP (mm Hg)	62 ± 5	62 ± 6	0.654	0.15
BRS (ms mm Hg^−1^)	13.3 [6.1]	10.8 [9.6]	0.297	0.45
RMSSD (ms)	61 [29]	47 [15]*	0.039	0.74
Ptot (ms^2^)	3867 [3348]	2449 [3052]	0.313	0.40
VLF (ms^2^)	1653 ± 1394	1083 ± 902	0.291	0.49
LF (ms^2^)	1529 ± 1254	1413 ± 1131	0.790	0.10
HF (ms^2^)	1062 [1775]	590 [769]	0.945	0.05
LFnu (%)	45.0 ± 20.8	52.2 ± 19.7	0.213	0.36
HFnu (%)	45.5 ± 20.6	39.5 ± 18.7	0.225	0.30
LF/HF	1.38 ± 1.13	1.94 ± 1.66	0.164	0.39

*P < 0.05 versus NH.

DAP, diastolic arterial blood pressure; nu, normalized unit; SAP, systolic arterial blood pressure.

**TABLE 2:** Parameters during incremental exercise in NH and HH.

	Exercise Intensity	Condition		
		NH	HH	Effect Size
Power output^C, I, C × I^ (W)	${\dot{W}}_{60/80}$	73 ± 10	73 ± 10	—
	${\dot{W}}_{165/200}$	187 ± 18	187 ± 18	—
	${\dot{W}}_{\max}$	238 ± 40	246 ± 38**	0.20
SpO_2_^C, I^ (%)	${\dot{W}}_{60/80}$	76.7 ± 2.2	78.2 ± 2.1*	0.70
	${\dot{W}}_{165/200}$	74.5 ± 2.3	75.8 ± 1.2*	0.71
	${\dot{W}}_{\max}$	74.9 ± 2.0	76.4 ± 2.0*	0.73
HR^C, I^ (min^−1^)	${\dot{W}}_{60/80}$	122 ± 17	121 ± 15	0.06
	${\dot{W}}_{165/200}$	168 ± 13	165 ± 15	0.20
	${\dot{W}}_{\max}$	180 ± 7	180 ± 7	0.05
$\dot{V}O_{2}$^I, C × I^ (L·min^−1^ stpd)	${\dot{W}}_{60/80}$	1.428 ± 0.129	1.390 ± 0.163	0.25
	${\dot{W}}_{165/200}$	2.505 ± 0.292	2.472 ± 0.289	0.12
	${\dot{W}}_{\max}$	2.863 ± 0.460	3.041 ± 0.493**	0.37
$\dot{V}O_{2}/\mathrm{HR}$^I, C × I^ (mL·beat^−1^)	${\dot{W}}_{60/80}$	11.9 ± 2.2	11.7 ± 2.2	0.12
	${\dot{W}}_{165/200}$	15.1 ± 2.6	15.2 ± 2.8	0.03
	${\dot{W}}_{\max}$	16.0 ± 3.0	16.9 ± 3.2***	0.31
RER^I, C × I^	${\dot{W}}_{60/80}$	0.89 ± 0.05	0.88 ± 0.03	0.18
	${\dot{W}}_{165/200}$	1.08 ± 0.05	1.04 ± 0.06**	0.69
	${\dot{W}}_{\max}$	1.18 ± 0.05	1.18 ± 0.05	0.02
${\dot{V}}_{E}$^I, C × I^ (L·min^−1^ btps)	${\dot{W}}_{60/80}$	42.8 ± 4.9	42.2 ± 6.5	0.12
	${\dot{W}}_{165/200}$	98.2 ± 17.6	95.1 ± 16.1	0.18
	${\dot{W}}_{\max}$	147.8 ± 34.8	164.1 ± 39**	0.44
V_T_^C, I^ (L btps)	${\dot{W}}_{60/80}$	1.62 ± 0.25	1.50 ± 0.23*	0.50
	${\dot{W}}_{165/200}$	2.51 ± 0.43	2.40 ± 0.41	0.27
	${\dot{W}}_{\max}$	2.62 ± 0.45	2.62 ± 0.43	0.00
*f*_R_^C, I, C × I^ (min^−1^)	${\dot{W}}_{60/80}$	27.0 ± 4.6	28.5 ± 4.9	0.33
	${\dot{W}}_{165/200}$	39.8 ± 8	40.5 ± 9.2	0.09
	${\dot{W}}_{\max}$	56.4 ± 8.9	62.6 ± 10.2***	0.65
${\dot{V}}_{E}/\dot{V}O_{2}$^I, C × I^ (btps/stpd)	${\dot{W}}_{60/80}$	30.0 ± 2.8	30.3 ± 2.9	0.09
	${\dot{W}}_{165/200}$	39.2 ± 5.1	38.5 ± 5.5	0.12
	${\dot{W}}_{\max}$	51.2 ± 5.6	53.5 ± 5.5*	0.41
${\dot{V}}_{E}/\dot{V}{\mathrm{CO}}_{2}$^I^ (btps/stpd)	${\dot{W}}_{60/80}$	33.8 ± 3.0	34.3 ± 2.5	0.18
	${\dot{W}}_{165/200}$	36.1 ± 4.1	36.8 ± 3.7	0.16
	${\dot{W}}_{\max}$	43.3 ± 4.9	45.2 ± 3.6	0.44
P_ET_O_2_^I, C × I^ (mm Hg)	${\dot{W}}_{60/80}$	51.1 ± 2.7	51.9 ± 2.6	0.27
	${\dot{W}}_{165/200}$	60.3 ± 3.1	60.3 ± 3.4	0.01
	${\dot{W}}_{\max}$	66.9 ± 2.7	68.4 ± 2.1*	0.61
P_ET_CO_2_^C, I, C × I^ (mm Hg)	${\dot{W}}_{60/80}$	35.1 ± 2.6	34.0 ± 2.0	0.46
	${\dot{W}}_{165/200}$	32.0 ± 3.5	30.9 ± 3.2	0.34
	${\dot{W}}_{\max}$	27.2 ± 2.2	25.2 ± 1.6*	1.07

^C^ significant effect of condition (HH vs NH), ^I^ significant effect of exercise intensity, ^C × I^ significant effect of condition–exercise intensity interaction.

*P < 0.05, **P < 0.01, and ***P < 0.001 versus NH.

nu, normalized unit; ${\dot{W}}_{60/80}$, 60 W for females and 80 W for males; ${\dot{W}}_{165/200}$, 165 W for females and 200 W for males; ${\dot{W}}_{\max}$, maximal power output.

**FIGURE 4:**
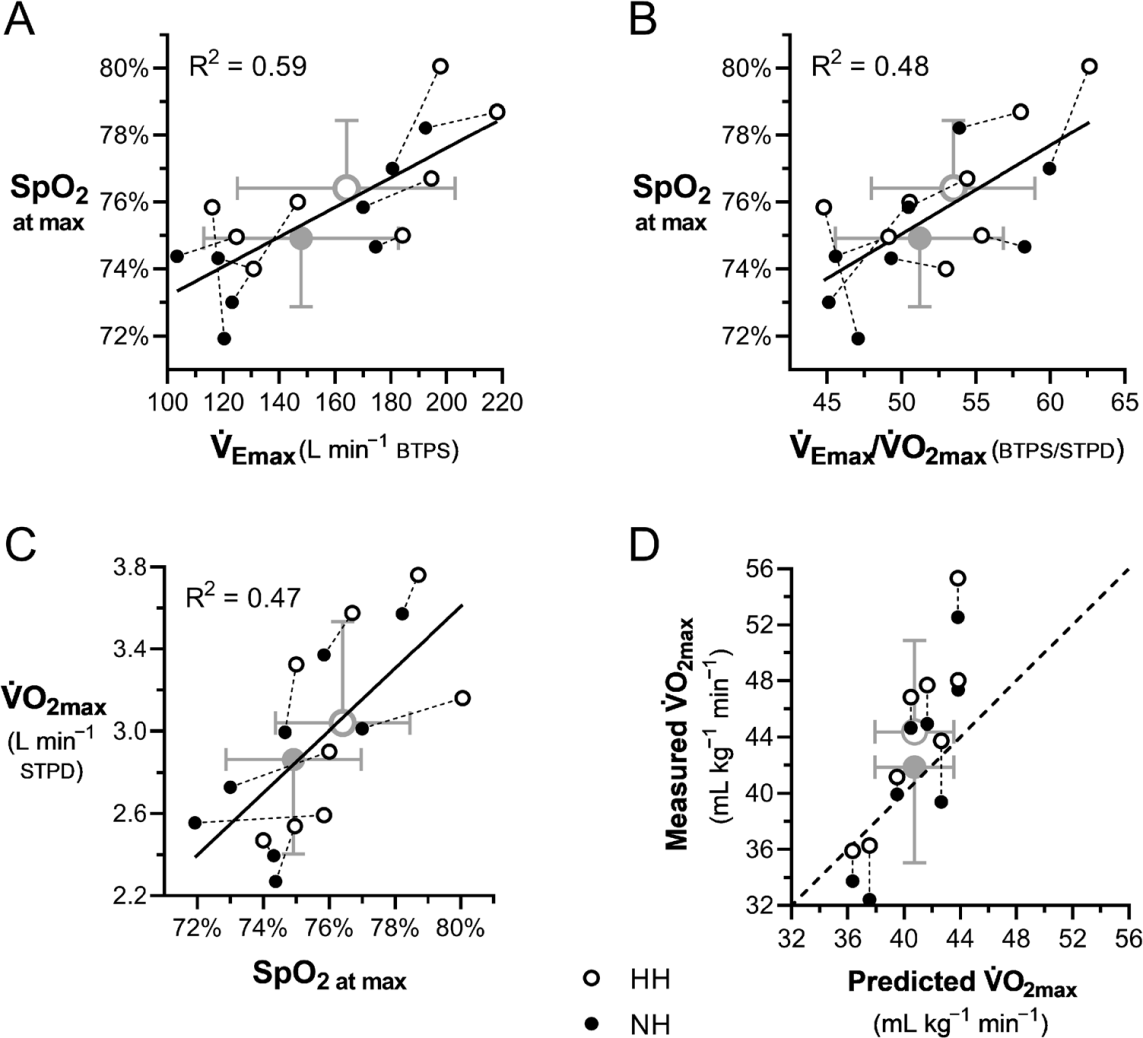
A and B, At maximal exercise, SpO_2_ increases as a function of maximal minute ventilation, both absolute ( ${\dot{V}}_{\text{Emax}}$) and normalized for maximal oxygen uptake ( ${\dot{V}}_{\text{Emax}}/\dot{V}O_{2\max}$). C, $\dot{V}O_{2\max}$ increases as a function of SpO_2_. D, Relationship between measured and predicted (21) $\dot{V}O_{2\max}$, with the dashed line representing the identity line. For all panels: open symbols, HH; closed symbols, NH; gray symbols, group means.

SpO_2_ at maximal exercise was positively related to ${\dot{V}}_{\text{Emax}}$, both absolute (Fig. 4A) and normalized for $\dot{V}O_{2\max}$ ( ${\dot{V}}_{\text{Emax}}/\dot{V}O_{2\max}$) (Fig. 4B). $\dot{V}O_{2\max}$ was positively related to SpO_2_ at maximal exercise (Fig. 4C). On average, the $\dot{V}O_{2\max}/\mathrm{kg}$ predicted from the meta-analytic equation (21) was lower (40.7 ± 2.8 mL·kg^−1^·min^−1^) than that measured in HH (44.4 ± 6.5 mL·kg^−1^·min^−1^, *P* = 0.046) but not in NH (41.9 ± 6.8 mL·kg^−1^·min^−1^, *P* = 0.508) and drifted from the identity line at high $\dot{V}O_{2\max}/\mathrm{kg}$ (Fig. 4D).

## DISCUSSION

The main findings of this study are that under fully controlled conditions and matched P_I_O_2_ corresponding to an equivalent altitude of ~4000 m, HH and NH elicit similar acute physiological responses at rest, but with increasing exercise intensity, differences in breathing pattern or mechanics occur, which can improve SpO_2_ and $\dot{V}O_{2\max}$ in HH.

### HH versus NH effects at rest

Our data support that respiratory differences at rest between short-term HH and NH exposure at an equivalent altitude of ~4000 m are minimal, if existent. The present findings are in agreement with Faulhaber et al. (18) but contrary to Savourey et al. (8,10), who showed lower SpO_2_ and arterial PO_2_ and PCO_2_ in HH than in NH. The latter findings may be explained by the timing of measurement, as measurements were performed during and immediately after a rapid decompression, when N_2_ exhalation in HH is substantial and can dilute alveolar gases (25). Given the small peak tissue N_2_ outflow of ~10 mL·min^−1^ btps^2^ and its ~24-min half-time (24), our 20-min decompression plus 20-min exposure to the target *P*_B_ likely ensured that resting alveolar N_2_ partial pressure was only ~0.3 mm Hg above, and resting P_A_O_2_ only ~0.2 mm Hg below, their respective steady-state values in HH. Therefore, it is not surprising that we observed similar resting P_ET_O_2_ and SpO_2_ between HH and NH (Table 1). Conversely, there are two ${\dot{V}}_{E}$-independent mechanisms by which resting P_ET_O_2_ and SpO_2_ could be even higher in HH than in NH: 1) the “alveolar gas equation effect” (27), which, however, yields a P_A_O_2_ gain in HH of only +0.2 ± 2.4 mm Hg when inserting the individual resting P_I_O_2_, P_ET_CO_2_, RER, and F_I_O_2_ into the equation, and 2) an increased gas diffusivity induced by hypobaria *per se* (35), which could enhance lung diffusion capacity, although this effect should also be minimal at rest according to our results.

The slightly lower HRV indices in HH compared with NH are in line with the results of Aebi et al. (9), although less pronounced (only RMSSD significantly differed between conditions in our study), probably because their hypoxic dose was slightly higher in HH than in NH (P_I_O_2_ 70 vs 74 mm Hg)(9). The effect of the breathing pattern can be ruled out as it was the same in HH and NH. Nevertheless, as respiratory sinus arrhythmia and HRV indices are positively related to intrathoracic pressure swings (36,37), the decrease in intrathoracic pressure swings by the lower air density may be a plausible mechanism for the lower HRV in in HH.

### HH versus NH effects during maximal exercise

The agreement between peak HR and $\dot{V}O_{2}$ during incremental exercise and the verification bout support maximal effort in both conditions. These data show that at ~4000 m equivalent altitude, $\dot{V}O_{2\max}$ was +6% ± 4% higher in HH than in NH (*P* < 0.01), with a decline from normoxia of −20% ± 3% in HH versus −24% ± 5% in NH (*P* < 0.001). The increase in ${\dot{V}}_{\text{Emax}}$ from NH to HH was even more pronounced (+11% ± 8%, *P* < 0.01 vs percent difference in $\dot{V}O_{2\max}$), resulting in an increase in the ${\dot{V}}_{\text{Emax}}/\dot{V}O_{2\max}$ ratio. These findings (Fig. 4A–C) are consistent with previous reports, where a higher ${\dot{V}}_{E}$ for a given $\dot{V}O_{2}$ has been consistently associated with a higher SpO_2_ and $\dot{V}O_{2\max}$ in ambient hypoxia (20,38,39), as the operating point lies on the steep part of the hemoglobin dissociation curve (19). As a result, the meta-analytic prediction equation of Macinnis et al. (21) agreed with our group average $\dot{V}O_{2\max}$ in NH, but systematically underestimated it in HH, with poor agreement at the individual level in both conditions (Fig. 4D). Therefore, caution should be exercised when using regression equations to predict individual $\dot{V}O_{2\max}$ decline in acute hypoxia, especially in HH.

Interestingly, the higher $\dot{V}O_{2\max}$ in HH was not accompanied by a higher HR_max_ (Fig. 3B), suggesting that it was not associated with a greater sympathetic activation or vagal withdrawal (40). The resulting higher $\dot{V}O_{2\max}/{\mathrm{HR}}_{\max}$ ratio in HH (Fig. 3B) could be the consequence of a greater maximal arteriovenous O_2_ difference secondary to the greater arterial O_2_ content, but also of a higher maximal stroke volume resulting from the higher *f*_R_ (i.e., more respiratory pump cycles per unit of time). Another interesting finding is the constancy of maximal exercise V_T_ between HH and NH, with the increase in ${\dot{V}}_{\text{Emax}}$ entirely driven by *f*_R_ (Fig. 3A). This finding, however, cannot be easily interpreted within existing models for the differential control of *f*_R_ and V_T_, as these models mostly apply to normobaric normoxic environments (41).

### HH versus NH effects during submaximal exercise

Contrary to previous reports of unchanged or lower SpO_2_ in HH compared with NH during submaximal exercise (13,15,18), we found a slightly higher SpO_2_ in HH despite similar ${\dot{V}}_{E}$. However, studies reporting lower SpO_2_ in HH relied on natural altitude ascent or investigated prolonged exposure to altitude (13,15), where it is more difficult to match the time of exposure and environmental/psychological conditions.

The “alveolar gas equation effect” could partially explain SpO_2_ differences during moderate exercise ( ${\dot{W}}_{60/80}$), as RER was <1.0. In fact, inserting the individual moderate-exercise P_I_O_2_, RER, and P_ET_CO_2_ into the equation yields a P_A_O_2_ gain in HH of +1.1 ± 1.5 mm Hg (*P* = 0.078). Accordingly, the measured P_ET_O_2_ gain in HH during moderate exercise, although not statistically significant, was of similar magnitude and direction (+0.7 ± 1.4 mm Hg) and highly correlated with that of P_A_O_2_ (*R*^2^ = 0.94). The “alveolar gas equation effect” is, however, nil at the high-intensity step ( ${\dot{W}}_{165/200}$) as the RER was close to 1.0.

The reduced air density could have led to improved SpO_2_ in HH via several mechanisms other than an increase in ${\dot{V}}_{E}$ both during moderate- and high-intensity exercise. First, as discussed previously, the increased gas diffusivity in HH could have enhanced O_2_ diffusion (35). Second, the reduced work of breathing and diaphragm fatigue might have allowed a higher fraction of the cardiac output to be delivered to locomotor muscles (42), resulting in higher mixed venous O_2_ content and, at the same ventilation/perfusion ratio, arterial O_2_ content. Third, the reduced airway resistance, as well as our observed lower V_T_ compared with NH, could attenuate intrathoracic pressure swings in HH, which has been demonstrated to decrease venous return and cardiac output (43). Since lung diffusion limitation (44) and right-to-left shunt (45) occur even during light exercise in ambient hypoxia, a reduced cardiac output is theoretically beneficial for SpO_2_ in this setting due to the increase in lung capillary transit time and reduced shunt blood flow. Interestingly, hypobaria *per se*, both in normoxia and hypoxia, was associated with lower cardiac output at 100 W and lower intrapulmonary shunt at any exercise intensity (46). The effect of air density on the work of breathing, and therefore on the competition for blood flow between respiratory and locomotor muscles, is even more important during high-intensity exercise (42). At this intensity, O_2_ delivery becomes critical, and further small impairments in locomotor muscle perfusion enhance anaerobic lactic metabolism (47). This was the case in the ${\dot{W}}_{165/200}$ step, where RER was higher in NH compared with HH, while ${\dot{V}}_{E}/\dot{V}{\mathrm{CO}}_{2}$ was similar, indicating greater lactic acidosis in NH. It is worth noting that, due to the higher $\dot{V}O_{2\max}$ in HH, ${\dot{W}}_{165/200}$ corresponded to 82% ± 8% of $\dot{V}O_{2\max}$ in HH and 88% ± 7% in NH (*P* < 0.001), and when this is accounted for, the differences in RER disappear, whereas those in ventilation are amplified (Fig. 2). Thus, hyperventilation was higher for a given level of metabolic acidosis in HH compared with NH. The acidosis-induced rightward shift of the hemoglobin dissociation curve may therefore be an additional explanation for the lower SpO_2_ during the ${\dot{W}}_{165/200}$ step in NH.

### Methodological considerations

The present study has several methodological strengths. Unlike others (8,10,14), we did not use respiratory valves and tubing systems, ensuring minimal perturbation of the ventilatory pattern, while still meticulously matching all environmental conditions, particularly P_I_O_2_. Furthermore, our measurements followed a fixed and consistent timing from the start of the simulated ascent, allowing for significant tissue N_2_ wash-in/washout. This contrasts with other studies that focused on the ascent itself and the immediately following period (8,14) or employed different timing of hypoxic exposure/acclimatization between HH and NH at the start of measurements (13,15,48). Finally, subject blinding, randomization, and crossover ensured the lowest possible risk of bias.

This study also has some limitations. First, the low number of subjects may have reduced the statistical power, although this could have been mitigated by the methodological strengths mentioned previously. Second, despite the precautions taken, motion artifacts may still have affected the accuracy of fingertip SpO_2_. Third, the time to exhaustion of the 105% ${\dot{W}}_{\max}$ verification bout was at the lower limit of what is considered capable of eliciting HR_max_ and $\dot{V}O_{2\max}$, approximately 2 min (49,50). However, this should not be attributed to an excessive percentage of ${\dot{W}}_{\max}$, but rather to the relatively intense warm-up. In fact, when a verification bout was performed without a warm-up in acute hypoxia, a longer time to exhaustion was achieved even at 110% of ${\dot{W}}_{\max}$ (51). Finally, this study applies to healthy, motivated, and relatively fit subjects. Therefore, caution should be taken when applying these results to unfit subjects or patient groups, who do not reach such high ${\dot{V}}_{E}$ at exhaustion, and therefore may experience smaller differences between HH and NH at maximal exercise. On the other hand, it can be speculated that the gain in HH may be even higher in clinical populations with increased airway resistance who may be more sensitive to respiratory muscle unloading (52).

## CONCLUSIONS

In acute HH and NH equivalent to ~4000 m altitude, resting parameters were essentially similar, whereas exercise SpO_2_ was slightly but systematically higher in HH compared with NH. At maximal exercise, the higher ${\dot{V}}_{\text{Emax}}$ in HH (driven by *f*_R_) likely contributed to this, which also led to a higher $\dot{V}O_{2\max}$. During submaximal exercise, SpO_2_ improved in HH by mechanisms other than increased ${\dot{V}}_{E}$, possibly because relative exercise intensity was lower compared with NH, and at the same relative exercise intensity, hyperventilation was delayed in NH compared with HH. These findings are likely due to respiratory muscle unloading in HH, which operated through different mechanisms depending on exercise intensity.

## Footnotes

^1^

$P_{A}O_{2}=P_{I}O_{2}-\frac{P_{A}{\mathrm{CO}}_{2}}{\mathrm{RER}}+F_{I}O_{2}\cdot P_{A}{\mathrm{CO}}_{2}\cdot \frac{1-\mathrm{RER}}{\mathrm{RER}}$

^2^
Resulted converting to our P_B_ the value of 15.45 mL·min^−1^ reported in (24), which we assume to be at body temperature and pressure, dry as reported in the methodological paper of the same research group (53).
